# The influence of the IKAP nursing model on wound healing following vacuum sealing drainage in acute infective endocarditis: a retrospective study

**DOI:** 10.3389/fcvm.2026.1671655

**Published:** 2026-03-11

**Authors:** Xichun Zhang, Yingli Shi, Yan Yang

**Affiliations:** 1 Department of Cardiovascular Medicine I, NanYang First People’s Hospital, Nanyang, Henan, China; 2 Department of Cardiac and Large Vascular Surgery, NanYang First People’s Hospital, Nanyang, Henan, China

**Keywords:** acute infectiveEndocarditis, IKAP nursing model, retrospective study, vacuum sealing drainage, wound healing

## Abstract

**Background:**

Acute infective endocarditis (IE) presents clinical challenges due to its complex pathophysiology and potential for severe complications. Vacuum sealing drainage (VSD) is an essential treatment approach, and nursing care plays a pivotal role in patient outcomes. The Innovative Knowledge-Attitude-Practice (IKAP) nursing model is a patient-centered approach emphasizing health education, behavioral engagement, and self-management support. This study aimed to investigate the association between the IKAP nursing model and wound healing following VSD in patients with acute IE.

**Methods:**

A retrospective analysis of 240 patients with acute IE undergoing VSD was conducted from January 2023 to December 2023. Patients were categorized into routine nursing (*n* = 117) and IKAP nursing (*n* = 123) groups based on admission period. Baseline characteristics, VSD details, laboratory results, medication usage, hospitalization details, wound size reduction, pain scores, and postoperative complications were compared. Multivariate logistic regression was performed to adjust for potential confounders.

**Results:**

Baseline disease-related characteristics, VSD characteristics, and several laboratory parameters did not significantly differ between the two nursing groups. The IKAP nursing group demonstrated a lower white blood cell count (9.94 ± 2.29 vs. 10.57 ± 2.14 × 10^9^/L, *P* = 0.029), greater wound size reduction at week 4 (3.52 ± 0.82 vs. 3.77 ± 0.95 cm^2^, *P* = 0.034), lower pain scores at week 4 (5.45 ± 1.93 vs. 5.98 ± 1.85, *P* = 0.031), and reduced incidence of wound infection (0.81% vs. 6.84%, adjusted OR = 0.11, 95% CI: 0.01–0.89, *P* = 0.034) and other adverse events (1.63% vs. 8.55%, adjusted OR = 0.18, 95% CI: 0.04–0.84, *P* = 0.031) following VSD. However, these differences were not sustained at week 8.

**Conclusion:**

The findings suggest a potential association between the IKAP nursing model and improved wound healing, pain management, and reduced postoperative complications in patients undergoing VSD for acute IE, particularly during the early recovery phase. Given the retrospective nature and observed attenuation of effects at week 8, prospective studies are warranted to confirm these findings.

## 1 Introduction

Acute infective endocarditis (IE) is a life-threatening infection of the endocardium, often involving the heart valves (1, 2). Its management is challenging due to complex pathophysiology and the potential for severe complications, including valvular destruction, heart failure, systemic embolization, and stroke. Effective and timely intervention is essential to prevent irreversible damage to cardiac structures and other organs (2, 3).

Vacuum sealing drainage (VSD), also known as vacuum-assisted closure (VAC), has emerged as an important adjunct in the treatment of patients with IE, particularly those with complex or infected wounds (4, 5). VSD involves applying controlled negative pressure to the wound surface, thereby promoting granulation tissue formation, enhancing drainage, and accelerating wound healing. It has been associated with improved healing times, reduced infection risk, and better postoperative recovery in various surgical and infectious scenarios, including acute IE (6–8).

In parallel, nursing care plays a critical role in optimizing outcomes for patients with IE. Beyond routine wound care, nursing interventions that emphasize patient education, communication, and behavioral support can significantly influence recovery trajectories. In this context, the Innovative Knowledge-Attitude-Practice (IKAP) nursing model offers a structured approach to enhancing patient engagement and self-care. The IKAP model focuses on improving patients' understanding of their condition, fostering positive health attitudes, and reinforcing proper self-management behaviors through individualized education and communication strategies (9–11). By empowering patients to participate actively in their care, the IKAP model may contribute to better wound healing, pain control, and reduction in postoperative complications.

Given the multifaceted needs of patients with acute IE, especially during VSD treatment, evaluating the effectiveness of such nursing interventions is essential. Therefore, this study aimed to investigate the association between the IKAP nursing model and wound healing, pain, and complication rates in patients with acute infective endocarditis undergoing vacuum sealing drainage.

## 2 Materials and methods

### 2.1 Study population

This retrospective study analyzed 240 patients with acute IE who underwent VSD and were admitted to Nanyang First People's Hospital between January 2023 and December 2023. Patients admitted between January and June 2023 received routine nursing care (*n* = 117), while patients admitted between July and December 2023 received IKAP nursing care (*n* = 123) following the implementation of the IKAP nursing protocol at our institution. This time-based allocation was non-randomized and based on the sequential implementation of the IKAP model in clinical practice. To address potential confounding arising from this non-randomized design, we employed multivariate regression analysis adjusting for baseline demographic and clinical characteristics including age, gender, BMI, hypertension, diabetes mellitus, coronary artery disease, congestive heart failure, and chronic kidney disease.

### 2.2 Inclusion and exclusion criteria

Inclusion criteria (12): (1) A diagnosis of acute IE and treatment with VSD; (2) Age ≥ 18 years; (3) Having normal cognitive function and complete, authentic, and valid clinical diagnostic data.

Exclusion criteria: (1) Allergy to the drugs used in this study; (2) Coexisting psychiatric disorders; (3) Pregnancy or lactation; (4) Severe hepatic or renal dysfunction.

### 2.3 Nursing model

#### 2.3.1 Traditional nursing

Traditional nursing care involves gathering basic patient information such as name, age, and condition, conveying physician instructions, conducting regular and timely ward rounds, and monitoring patient vital signs and treatment effectiveness according to the prescribed protocols.

#### 2.3.2 IKAP nursing

Patients received comprehensive IKAP nursing alongside traditional care. The IKAP model consisted of four key components: (1) Information provision through comprehensive admission assessment evaluating physiological and psychological condition, with thorough explanation of the illness and factors impacting quality of life; (2) Knowledge enhancement through disease-related information customized to each patient's comprehension level, using accessible language and multimedia tools (health education manuals, relevant videos) to explain causes, treatment methods, complications, prognosis, and nursing approaches; (3) Attitude modification by utilizing successful recovery cases and patient discussion groups to instill confidence, address negative emotions stemming from limited disease knowledge, and motivate active engagement in the clinical journey; and (4) Practice reinforcement through guidance on disease complications, adverse treatment reactions, and coping strategies to enhance self-care abilities, alongside structured follow-up via telephone and home visits. Patient and family involvement in care planning and supervision was actively encouraged throughout (9–11).

### 2.4 Data collection

Demographic data of patients, including age, gender, BMI, hypertension, diabetes, educational and employment status, and housing type, were collected from the medical record system. Baseline disease-related characteristics such as coronary artery disease, congestive heart failure, valvular heart disease, chronic kidney disease, and chronic obstructive pulmonary disease were documented for each patient. Statistical analysis was conducted on the VSD details, including average time to drain removal and average daily drainage volume. Laboratory results, comprising white blood cell count, C-reactive protein levels, erythrocyte sedimentation rate, procalcitonin, and troponin I levels, were collected and compared. Levels of inflammatory markers, including TNF-α, IL-6, IL-10, MCP-1, and VCAM-1, were also recorded and compared between the two groups. Medication details, encompassing antibiotics, analgesics, anticoagulants, antiarrhythmic drugs, and vasoactive agents, were documented for both patient groups. Hospitalization details and surgical complications such as length of hospital stay, surgical site infections, deep vein thrombosis, pulmonary embolism, and acute kidney injury were recorded for statistical analysis. Additionally, wound reduction data consisting of initial wound size and wound size at the 4th and 8th weeks, as well as pain scores before nursing care and at 4 and 8 weeks into nursing care, were collected. Postoperative complications and adverse reactions, including wound infections, bleeding events, allergic reactions, hypotension, and other adverse events, were also recorded for analysis.

### 2.5 Blood tests

A fasting venous blood sample of 5 mL was collected at 8 AM for the following tests. White blood cell count (×10^9^/L) was examined using the Beckman Coulter's DxH800 Hematology Analyzer. C-reactive protein levels (mg/L) were determined using the BECKMAN Synchron lx20 automatic biochemical analyzer with a rate nephelometry method. Whole blood anticoagulated with ethylenediaminetetraacetic acid (EDTA) was used for erythrocyte sedimentation rate (ESR, mm/h) determination, performed using the TEST1 automatic erythrocyte sedimentation rate analyzer. Troponin I (ng/mL) was measured using the ELISA method, with reagents provided by Nanjing KeyGen Biotech Co., Ltd., and absorbance values measured using the Bio-Tek Instruments, Inc. ELX800 microplate reader. For procalcitonin (PCT, ng/mL), TNF-α (pg/mL), IL-6 (pg/mL), IL-10 (pg/mL), MCP-1 (pg/mL), and VCAM-1 (ng/mL) detection, the supernatant was obtained after centrifugation at 3,000 rpm for 5 min and subjected to enzyme-linked immunosorbent assay (ELISA) using specific assay kits: PCT (ab221828, Abcam, USA), TNF-α (ab181421, Abcam, USA), IL-6 (ab178013, Abcam, USA), IL-10 (ab185986, Abcam, USA), MCP-1 (ab179886, Abcam, USA), and VCAM-1 (ab47355, Abcam, USA).

### 2.6 Wound measurement

All wounds were measured and photographed by the same physician before care, after 4 weeks of care, and at 8 weeks of care, always with the same photography equipment. Wound dimensions were assessed using a sterile ruler and Vernier caliper (MEB-6/150). Digital photographs were analyzed using ImageJ software (National Institutes of Health, USA) for surface area and perimeter calculations.

### 2.7 Visual analogue scale (VAS) scores

Pain was assessed using the Visual Analogue Scale (VAS), a validated self-reported tool with a reported reliability coefficient of 0.94 (13). Patients rated their pain intensity on a numerical scale ranging from 0 to 10, where 0 denoted no pain, scores of 1 to 3 indicated mild pain, 4 to 6 signified moderate pain that could potentially disrupt sleep but was still bearable, and 7 to 10 represented severe or intense pain significantly impacting sleep quality and appetite.

### 2.8 Statistical methods

Data were analyzed using SPSS 29.0 statistical software (SPSS Inc., Chicago, IL, USA). The Shapiro–Wilk test was used to assess normal distribution. Data confirmed to follow normal distribution were described as mean ± standard deviation (x¯ ± s), and independent samples *t*-tests were used for between-group comparisons. For non-normally distributed data, the Mann–Whitney *U*-test was employed. Categorical data were analyzed using chi-squared tests or Fisher's exact test when expected cell counts were less than 5. Repeated measures analysis of variance was used for variables with multiple time points. To adjust for potential confounders arising from the non-randomized design, multivariate logistic regression was performed for binary outcomes and multivariate linear regression for continuous outcomes, adjusting for age, gender, BMI, hypertension, diabetes mellitus, coronary artery disease, congestive heart failure, and chronic kidney disease. Results are presented as adjusted odds ratios (OR) with 95% confidence intervals (CI) for categorical outcomes. *P* < 0.05 was considered statistically significant.

## 3 Results

### 3.1 Baseline characteristics

A total of 240 participants with acute IE were enrolled, with 117 in the routine nursing group and 123 in the IKAP nursing group (Table 1). The baseline characteristics, including age, gender distribution, BMI, prevalence of hypertension, diabetes mellitus, educational level, employment status, and residence type, were balanced between the two groups, with no statistically significant differences observed (*P* > 0.05). No significant differences were observed, confirming comparability between groups.

**Table 1:** Baseline characteristics of study participants.

Characteristics	Routine nursing (*n* = 117)	IKAP nursing (*n* = 123)	t/*χ*²	*P*
Age (years)	58.4 ± 6.72	59.13 ± 7.24	0.808	0.420
Gender (Male)	64 (54.7%)	72 (58.54%)	0.220	0.639
BMI (kg/m²)	24.85 ± 3.42	25.14 ± 3.12	0.683	0.495
Hypertension	48 (41.03%)	52 (42.28%)	0.004	0.948
Diabetes Mellitus	35 (29.91%)	38 (30.89%)	0.001	0.980
Educational Level	–	–	0.152	0.927
High School	45 (38.46%)	49 (39.84%)		
College	37 (31.62%)	40 (32.52%)		
University	35 (29.91%)	34 (27.64%)		
Employment (Employed)	63 (53.85%)	72 (58.54%)	0.362	0.547
Residence (Urban)	75 (64.1%)	75 (60.98%)	0.135	0.714

### 3.2 Baseline disease-related features

The distribution of coronary artery disease, congestive heart failure, valvular heart disease, chronic kidney disease, and chronic obstructive pulmonary disease showed no statistically significant differences between the two groups (*P* > 0.05), indicating similar baseline characteristics at the outset of the study (Table 2). These findings confirm that the groups were well-matched in terms of baseline disease-related features.

**Table 2:** Baseline disease-related features of study participants.

Parameters	Routine nursing (*n* = 117)	IKAP nursing (*n* = 123)	*χ*²	*P*
Coronary Artery Disease	54 (46.15%)	58 (47.15%)	0.001	0.979
Congestive Heart Failure	40 (34.19%)	45 (36.59%)	0.064	0.800
Valvular Heart Disease	21 (17.95%)	25 (20.33%)	0.092	0.762
Chronic Kidney Disease	36 (30.77%)	39 (31.71%)	0.000	0.986
COPD	17 (14.53%)	20 (16.26%)	0.037	0.848

COPD, chronic obstructive pulmonary disease.

### 3.3 VSD characteristics

**Figure 1:**
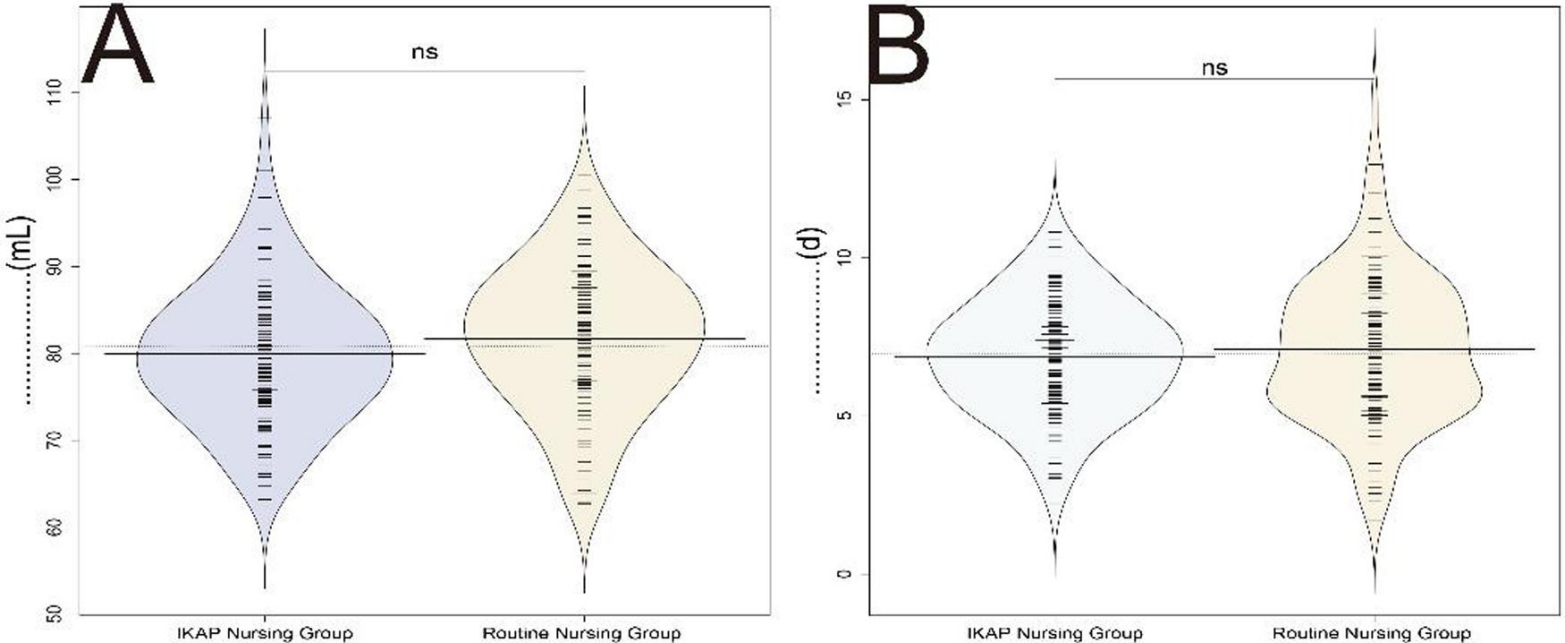
**(A)** Average tube removal time, **(B)** Average daily drainage volume.

In the investigation of VSD characteristics among patients undergoing treatment for acute IE, the mean extubation time and mean daily drainage volume were assessed in both groups (Figure 1). The results revealed no statistically significant differences between the two groups for either parameter (*t* = 0.958, *P* = 0.339 for mean extubation time; *t* = 1.656, *P* = 0.099 for mean daily drainage volume). Although not statistically significant, the trend toward reduced drainage volume in the IKAP group may warrant further investigation. These findings demonstrate similar VSD characteristics in the two nursing models.

### 3.4 Laboratory results

**Figure 2:**
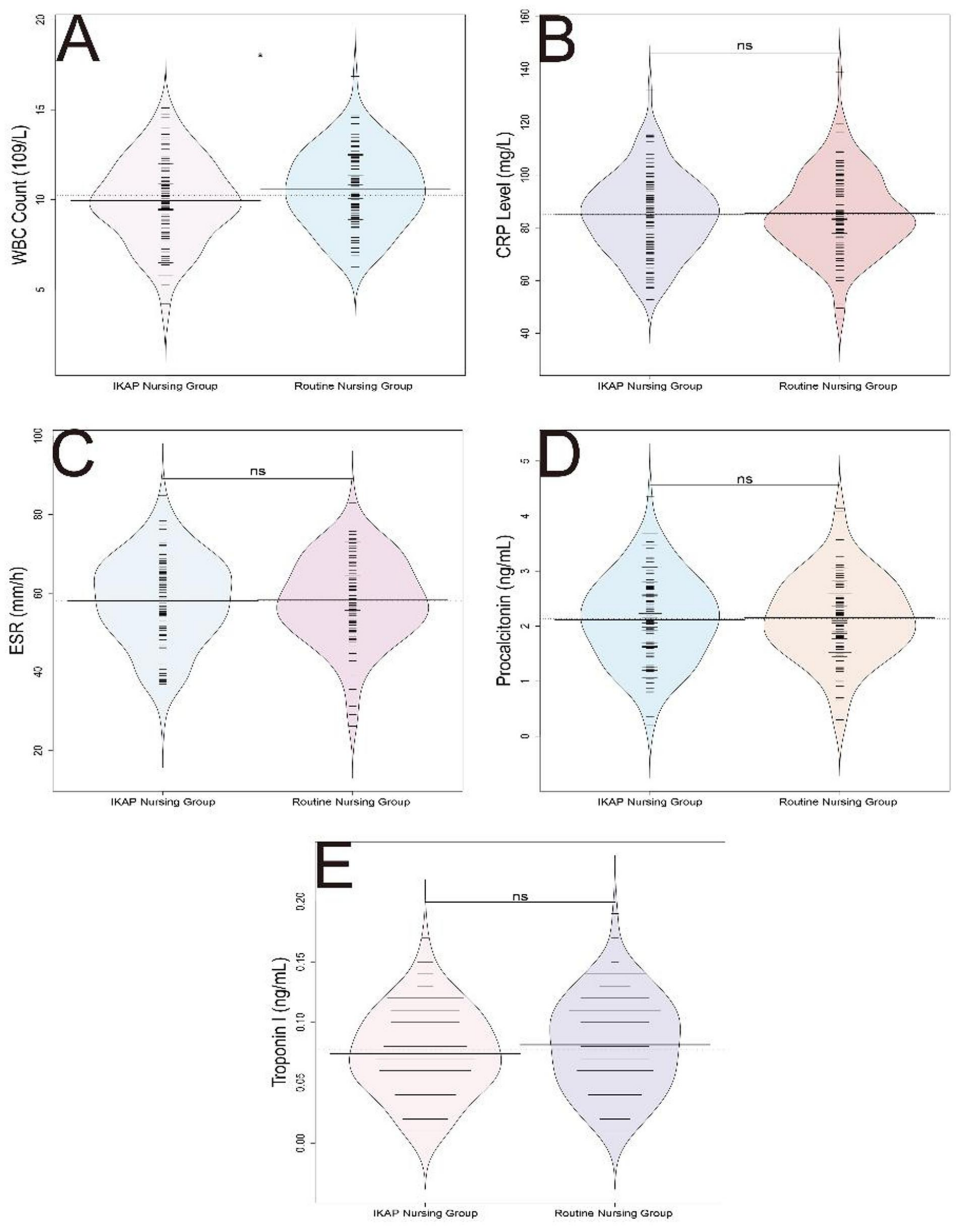
Comparison of laboratory results between routine nursing group and IKAP nursing group. **(A)** WBC count (109/L), **(B)** CRP level (mg/L), **(C)** ESR (mm/hj), **(D)** Procalcitonin (ng/mL), **(E)** Troponin I (ng/mL).

A statistically significant difference in white blood cell (WBC) count was observed between the two groups (*P* = 0.029), with a mean of 10.57 ± 2.14 × 10^9^/L in the routine nursing group and 9.94 ± 2.29 × 10^9^/L in the IKAP nursing group (t = 2.201). However, no statistically significant differences were observed for the levels of C-reactive protein (CRP), erythrocyte sedimentation rate (ESR), procalcitonin, and troponin I levels between the two nursing groups (*P* > 0.05) (Figure 2). The clinical significance of the isolated WBC difference remains uncertain given the lack of corresponding changes in other inflammatory markers.

### 3.5 Inflammatory markers

**Figure 3:**
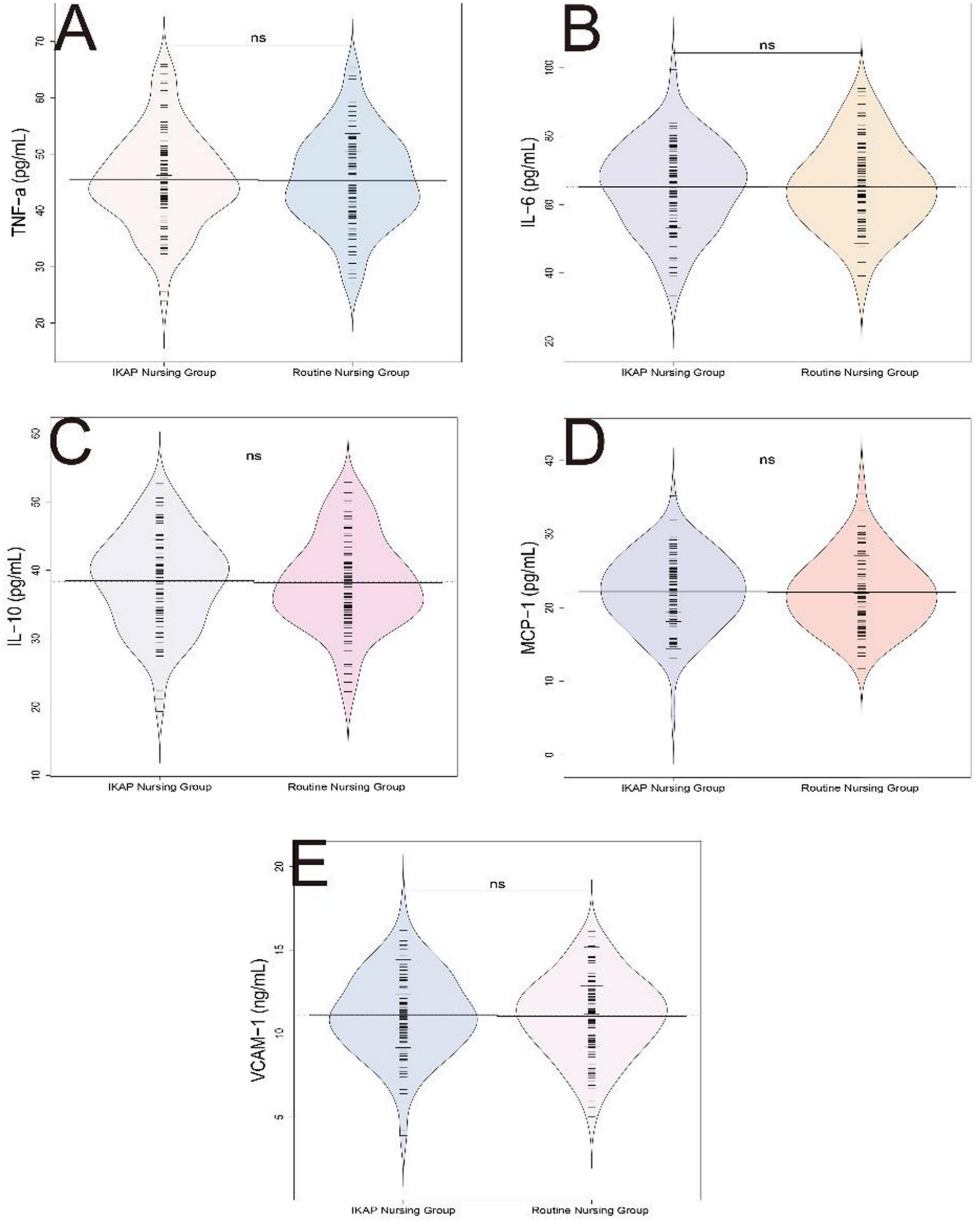
Comparison of inflammatory markers between routine nursing group and IKAP nursing group. **(A)** (/bd) e-NI, **(B)** IL-6 (7wybd), **(C)** IL-10 (pg/mL), **(D)** MCP-1 (pg/mL), **(E)** VCAM-1 (ng/mL).

Inflammatory marker levels measured postoperatively showed no significant intergroup differences. Specifically, no statistically significant differences were observed in the levels of tumor necrosis factor alpha (TNF-α) (45.36 ± 8.19 pg/mL vs. 45.42 ± 8.35 pg/mL, *t* = 0.054, *P* = 0.957), interleukin-6 (IL-6) (65.28 ± 12.45 pg/mL vs. 65.14 ± 12.59 pg/mL, *t*= 0.083, *P* = 0.934), interleukin-10 (IL-10) (38.12 ± 6.75 pg/mL vs. 38.48 ± 6.92 pg/mL, *t* = 0.404, *P* = 0.687), monocyte chemoattractant protein-1 (MCP-1) (22.13 ± 4.89 pg/mL vs. 22.18 ± 4.95 pg/mL, *t* = 0.079, *P* = 0.937), and vascular cell adhesion molecule-1 (VCAM-1) (11.04 ± 2.35 ng/mL vs. 11.12 ± 2.45 ng/mL, *t* = 0.271, *P* = 0.787) (Figure 3). These results indicate similar inflammatory marker profiles in both nursing models, suggesting that the IKAP model may not exert substantial effects on systemic inflammatory responses as measured by these biomarkers.

### 3.6 Medication usage

Similar medication patterns suggest both groups received consistent clinical management. No statistically significant differences were found in the duration of antibiotic therapy (14.32 ± 4.05 days vs. 13.97 ± 3.78 days, *t* = 0.694, *P* = 0.488), the number of analgesic doses administered (24.57 ± 5.68 vs. 23.78 ± 5.42, *t* = 1.099, *P* = 0.273), the duration of anticoagulant therapy (10.75 ± 2.91 days vs. 10.45 ± 2.63 days, *t* = 0.836, *P* = 0.404), the duration of antiarrhythmic medication use (8.23 ± 1.97 days vs. 8.05 ± 1.82 days, *t* = 0.749, *P* = 0.455), and the duration of vasopressor administration (36.84 ± 9.57 h vs. 35.94 ± 9.21 h, *t* = 0.736, *P* = 0.463) (Table 3).

**Table 3:** Medication usage in routine nursing group and IKAP nursing group.

Parameters	Routine Nursing (*n* = 117)	IKAP Nursing (*n* = 123)	t	*P*
Antibiotics (days)	14.32 ± 4.05	13.97 ± 3.78	0.694	0.488
Analgesics (doses)	24.57 ± 5.68	23.78 ± 5.42	1.099	0.273
Anticoagulants (days)	10.75 ± 2.91	10.45 ± 2.63	0.836	0.404
Antiarrhythmics (days)	8.23 ± 1.97	8.05 ± 1.82	0.749	0.455
Vasopressors (hours)	36.84 ± 9.57	35.94 ± 9.21	0.736	0.463

### 3.7 Length of hospital stay and surgical complications

There were no significant differences in the length of hospital stay (14.62 ± 3.21 days vs. 14.48 ± 3.15 days, *t* = 0.336, *P* = 0.737) between the two groups. Furthermore, the incidence of surgical site infection (6.84% vs. 4.88%, *χ*^2^ = 0.138, *P* = 0.710), deep vein thrombosis (4.27% vs. 3.25%, *χ*^2^ = 0.006, *P* = 0.939), pulmonary embolism (3.42% vs. 2.44%, *χ*^2^ = 0.005, *P* = 0.946), and acute kidney injury (5.13% vs. 6.5%, *χ*^2^ = 0.032, *P* = 0.858) did not differ significantly between the two nursing groups (Table 4). Due to low event rates, these results should be interpreted cautiously.

**Table 4:** Length of hospital stay and surgical complications.

Parameters	Routine nursing (*n* = 117)	IKAP nursing (*n* = 123)	t/*χ*²	*P*
Length of Stay (days)	14.62 ± 3.21	14.48 ± 3.15	0.336	0.737
Surgical Site Infection	8 (6.84%)	6 (4.88%)	0.138	0.710
Deep Vein Thrombosis	5 (4.27%)	4 (3.25%)	0.006	0.939
Pulmonary Embolism	4 (3.42%)	3 (2.44%)	0.005	0.946
Acute Kidney Injury	6 (5.13%)	8 (6.5%)	0.032	0.858

### 3.8 Wound size reduction

**Figure 4:**
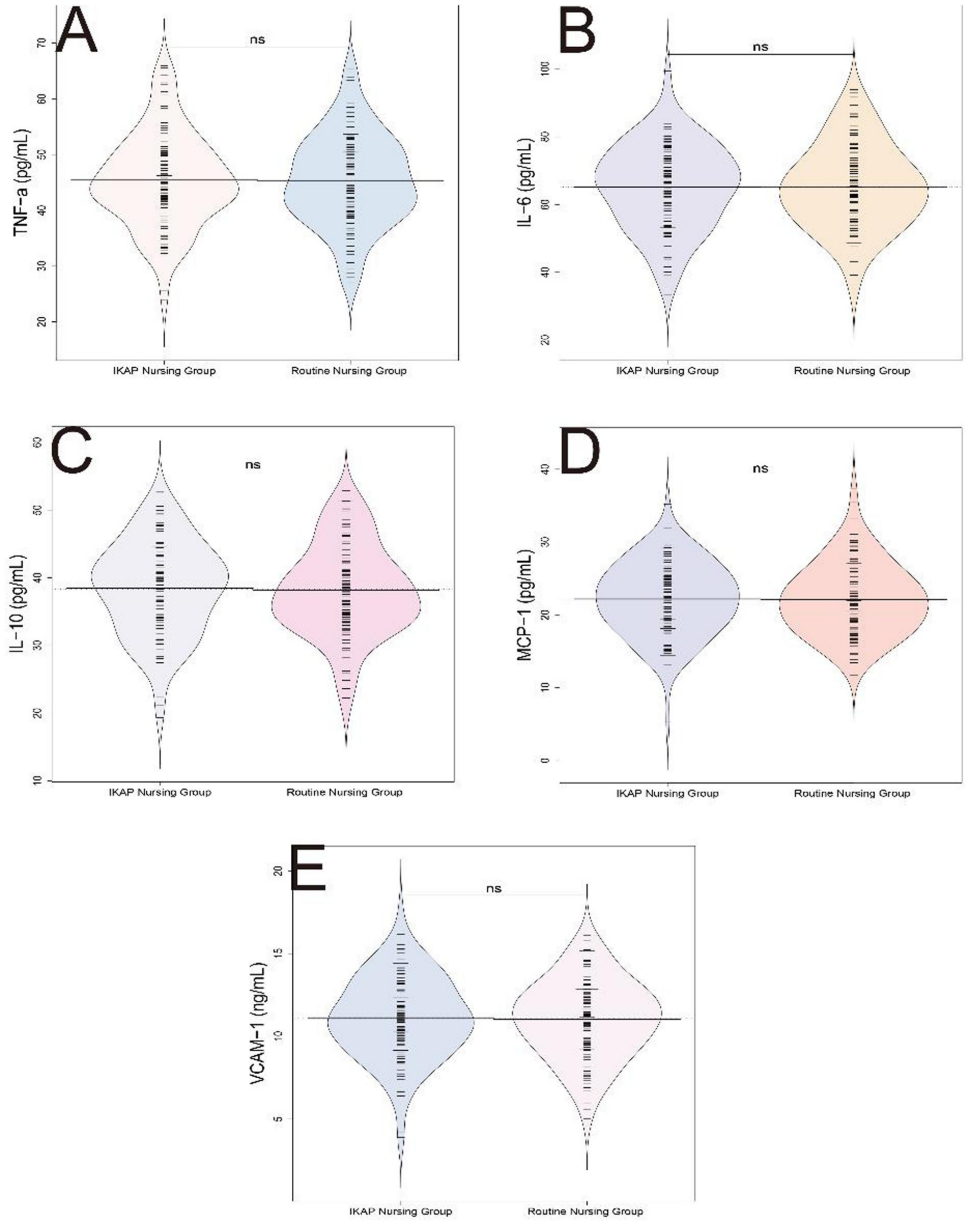
Comparison of wound size reduction. **(A)** TNF-a (pg/mL), **(B)** IL-6 (pg/mL), **(C)** IL-10 (pg/mL), **(D)** MCP-1 (pg/mL), **(E)** VCAM-1 (pg/mL).

In assessing wound size reduction in patients with acute IE undergoing VSD, the comparison between the routine nursing group and the IKAP nursing group revealed a statistically significant difference in wound size at week 4 (3.77 ± 0.95 cm^2^ vs. 3.52 ± 0.82 cm^2^, *t* = 2.133, *P* = 0.034). However, no statistically significant differences were observed in the initial wound size or the wound size at week 8 between the two nursing groups (*P* > 0.05) (Figure 4). The early reduction in wound size at week 4 may reflect improved initial wound management and patient engagement under the IKAP model. Notably, the effect was not sustained at week 8, which may suggest that the benefits of enhanced nursing engagement are most pronounced during the acute recovery phase when patient education and behavioral modification have their greatest impact, with subsequent convergence as natural wound healing processes predominate in the later recovery period.

### 3.9 Pain scores

**Figure 5:**
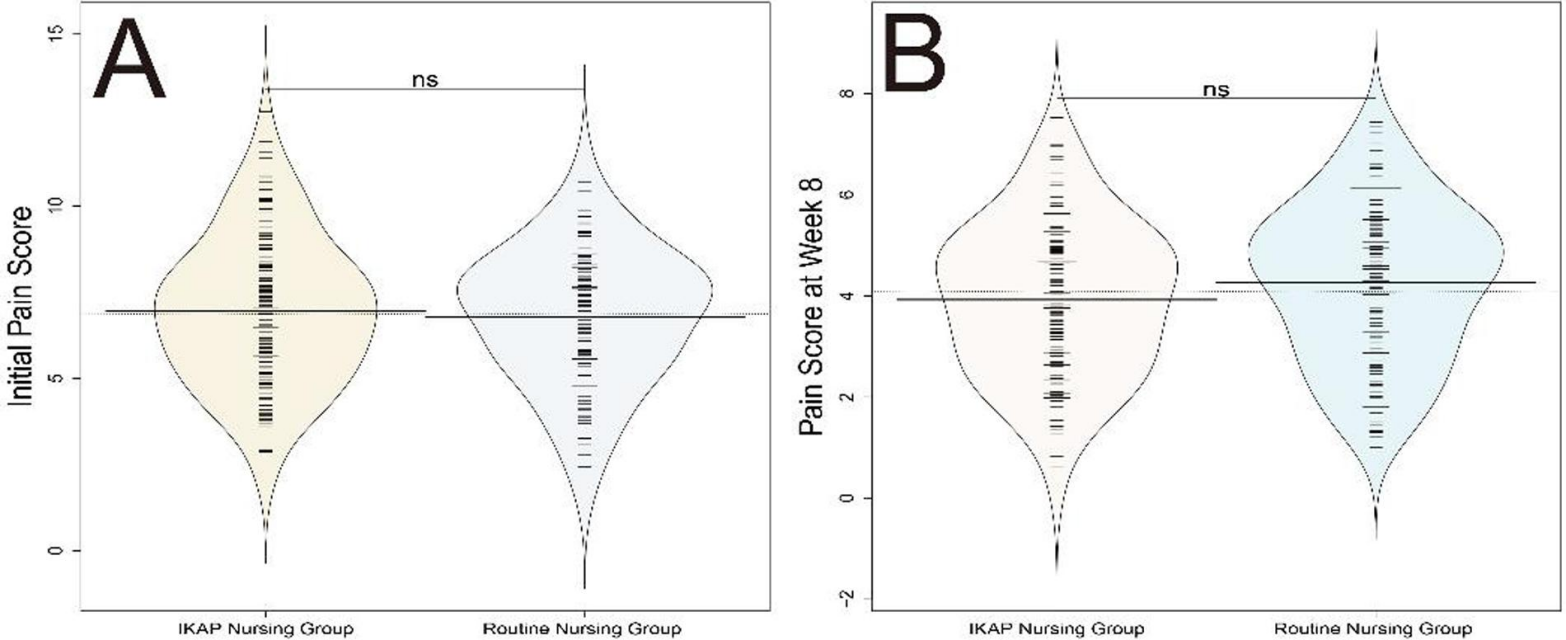
Comparison of pain scores between routine nursing and IKAP nursing groups. **(A)** Initial pain score, **(B)** Pain score at week 8.

In evaluating the pain scores of patients with acute IE undergoing VSD, a statistically significant difference was observed between the routine nursing group and the IKAP nursing group at week 4 (5.98 ± 1.85 vs. 5.45 ± 1.93, t = 2.168, *P* = 0.031). However, there were no significant differences in pain scores at baseline or at week 8 between the two groups (*P* > 0.05) (Figure 5). These findings suggest that the IKAP nursing model may have a positive association with pain reduction during the mid-phase of recovery. Similar to wound healing outcomes, the lack of sustained difference at week 8 indicates that the effect may be transient, possibly reflecting the time-limited impact of enhanced patient engagement and education on pain perception.

### 3.10 Postoperative complications and adverse reactions

In evaluating postoperative complications and adverse reactions among patients with acute IE undergoing VSD, significant differences were observed between the routine nursing group and the IKAP nursing group. The incidence of wound infection was significantly lower in the IKAP group compared to the routine care group (0.81% vs. 6.84%, *χ*^2^ = 4.476, *P* = 0.034), as was the incidence of other adverse events (1.63% vs. 8.55%, *χ*^2^ = 4.677, *P* = 0.031) (Table 5). After multivariate adjustment for age, gender, BMI, hypertension, diabetes mellitus, coronary artery disease, congestive heart failure, and chronic kidney disease, the associations remained significant (wound infection: adjusted OR = 0.11, 95% CI: 0.01–0.89; other adverse events: adjusted OR = 0.18, 95% CI: 0.04–0.84). However, there were no statistically significant differences between the groups in the incidence of bleeding events or allergic reactions (*P* > 0.05).

**Table 5:** Postoperative complications and adverse reactions.

Parameter	Routine (*n* = 117)	IKAP (*n* = 123)	*χ*²	*P*	Adjusted OR (95% CI)
Wound Infection	8 (6.84%)	1 (0.81%)	4.476	0.034	0.11 (0.01–0.89)
Bleeding Events	3 (2.56%)	2 (1.63%)	0.003	0.955	0.63 (0.10–3.87)
Allergic Reactions	4 (3.42%)	3 (2.44%)	0.005	0.946	0.71 (0.16–3.24)
Hypotension	2 (1.71%)	1 (0.81%)	0.002	0.965	0.47 (0.04–5.29)
Other Adverse Events	10 (8.55%)	2 (1.63%)	4.677	0.031	0.18 (0.04–0.84)

Adjusted for age, gender, BMI, hypertension, diabetes mellitus, coronary artery disease, congestive heart failure, and chronic kidney disease using multivariate logistic regression.

## 4 Discussion

Acute infective endocarditis (IE) presents a significant challenge in clinical management due to its complex pathophysiology and the potential for severe, multi-organ complications (14–16). IE involves infection of the endocardial surface of the heart, with clinical manifestations that may affect virtually every organ system. Cardiac complications such as valvular vegetations, myocardial abscesses, and myopericarditis are particularly serious and may require surgical intervention (17–19). Vacuum sealing drainage (VSD) has been widely adopted in the management of postoperative and infectious wounds due to its ability to promote effective drainage of both superficial and deep tissues, enhancing wound healing by reducing local edema, improving tissue perfusion, and promoting granulation tissue formation (20–22).

The present study sought to evaluate the association between the IKAP nursing model and postoperative recovery in patients with acute IE undergoing VSD. Our findings suggest that the IKAP model was associated with reduced incidence of wound infection and other adverse events, with a notable reduction in wound size and pain scores at week 4. However, these associations were not sustained at week 8 for wound and pain outcomes, and several important limitations warrant careful interpretation of these results.

A key finding was the observed difference in wound infection rates (0.81% vs. 6.84%, adjusted OR = 0.11) and other adverse events (1.63% vs. 8.55%, adjusted OR = 0.18) between the IKAP and routine nursing groups after multivariate adjustment. While statistically significant, the wide confidence intervals reflect the relatively small number of events and suggest uncertainty in the precise magnitude of effect. The clinical relevance of these findings requires confirmation in larger prospective studies.

The transient nature of the observed effects on wound size and pain scores merits discussion. The significant differences observed at week 4 but not at week 8 may reflect several possibilities. First, the IKAP model's emphasis on early patient education, engagement, and self-management may have its greatest impact during the acute recovery phase when patients are most receptive to behavioral modification and when nursing interventions can most directly influence care quality. Second, as wound healing progresses, natural healing processes may predominate, leading to convergence between groups. Third, the ceiling effect of wound healing progression may limit the detectable difference at later time points. These findings are consistent with prior studies suggesting that structured nursing interventions may be most beneficial during the early postoperative period (23–25).

Notably, while we observed a statistically significant difference in WBC count between groups, there were no corresponding differences in other inflammatory markers including TNF-α, IL-6, IL-10, MCP-1, and VCAM-1. This inconsistency limits our ability to draw conclusions about the IKAP model's effect on inflammatory responses. The isolated WBC finding may represent chance variation or may reflect aspects of the inflammatory response not captured by the other measured biomarkers. Future studies with more comprehensive inflammatory profiling and serial measurements would be needed to clarify any potential immunomodulatory effects of enhanced nursing care.

The IKAP nursing model's emphasis on comprehensive, tailored patient education and engagement may contribute to improved patient understanding of their illness, treatment methods, and self-care strategies (23–25). This enhanced knowledge and awareness may lead to greater adherence to medical recommendations, better self-management of symptoms, and reduced anxiety related to the disease and its management. The use of accessible language, health education materials, and patient discussion groups may empower patients to actively participate in their care (26–28). Additionally, the model's focus on patient advocacy, open communication, and proactive care discussions may contribute to optimized pain management and reduced vulnerability to complications (29, 30).

Several important limitations of this study must be acknowledged. First, the retrospective design precludes establishing causal relationships, and the observed associations may be influenced by unmeasured confounders despite multivariate adjustment. The time-based group allocation, while reflecting the practical reality of implementing a new nursing protocol, introduces potential temporal confounding including changes in clinical practices, staff experience, or patient populations over time. Second, the single-center nature of the study limits generalizability. Third, the lack of randomization raises concerns about selection bias, although baseline characteristics appeared balanced between groups. Fourth, we could not account for potential missing data or variations in data quality inherent to retrospective medical record review. Fifth, the relatively small sample size limits statistical power, particularly for detecting differences in rare complications, as reflected in the wide confidence intervals. Finally, the lack of long-term follow-up beyond 8 weeks prevents assessment of sustained benefits.

Future research should address these limitations through prospective, randomized, multi-center designs with adequate sample sizes and longer follow-up periods. Studies incorporating qualitative methods could provide insights into the mechanisms underlying any beneficial effects and the practical challenges of implementing the IKAP model. Cost-effectiveness analyses would help inform resource allocation decisions for healthcare systems considering adoption of enhanced nursing protocols.

## 5 Conclusion

The findings of this retrospective study suggest a potential association between the IKAP nursing model and improved wound healing, pain management, and reduced postoperative complications in patients undergoing VSD for acute IE, particularly during the early recovery phase. However, these associations were not sustained at week 8 for wound and pain outcomes, and the absence of differences in inflammatory biomarkers limits mechanistic interpretation. Given the inherent limitations of the retrospective design including potential selection bias and unmeasured confounding, these findings should be interpreted as hypothesis-generating. Prospective, randomized controlled trials are warranted to confirm these associations and establish causal relationships. If confirmed, these findings would support the integration of patient-centered, education-focused nursing models into routine cardiovascular postoperative care.

## Data Availability

The original contributions presented in the study are included in the article/Supplementary Material, further inquiries can be directed to the corresponding author.
